# A practical guide to botulinum neurotoxin treatment of shoulder spasticity 2: Injection techniques, outcome measurement scales, and case studies

**DOI:** 10.3389/fneur.2022.1022549

**Published:** 2022-12-07

**Authors:** Jörg Wissel, Alexandre Camões-Barbosa, Stefano Carda, Damon Hoad, Jorge Jacinto

**Affiliations:** ^1^Department of Neurorehabilitation and Physical Therapy, Vivantes Hospital Spandau, Berlin, Germany; ^2^Medicina Física e Reabilitação, Centro Hospitalar Universitário de Lisboa Central, Lisbon, Portugal; ^3^CHUV, Neuropsychology and Neurorehabilitation, Lausanne, Switzerland; ^4^Warwick Medical School, University of Warwick, Coventry, United Kingdom; ^5^Centro de Medicina de Reabilitação de Alcoitão, Serviço de Reabilitação de Adultos 3, Alcabideche, Portugal

**Keywords:** botulinum neurotoxin, muscle spasticity, shoulder, injection, pain, assessment tools

## Abstract

**Introduction:**

Botulinum neurotoxin type A (BoNT-A) is a first-line treatment option for post-stroke spasticity, reducing pain and involuntary movements and helping to restore function. BoNT-A is frequently injected into the arm, the wrist, the hand, and/or the finger muscles but less often into the shoulder muscles, despite clinical trials demonstrating improvements in pain and function after shoulder BoNT-A injection.

**Methods:**

In part 2 of this two-part practical guide, we present an experts' consensus on the choice of outcome measurement scales and goal-setting recommendations for BoNT-A in the treatment of shoulder spasticity to increase awareness of shoulder muscle injection with BoNT-A, alongside the more commonly injected upper limb muscles. Expert consensus was obtained from five European experts with a cumulative experience of more than 100 years of BoNT-A use in post-stroke spasticity. Case studies are included as examples of approaches taken in the treatment of shoulder spasticity.

**Results:**

Although the velocity-dependent increase in muscle tone is often a focus of patient assessment, it is only one component of spasticity and should be assessed as part of a wider range of measurements. For outcome measurement following BoNT-A injection in shoulder muscles, shoulder-specific scales are recommended. Other scales to be considered include Pain Numerical Rating and/or global functioning, as well as the quality of life and global perception of benefit scores.

Goal setting is an essential part of the multidisciplinary management process for spasticity; goals should be patient-centric, realistic, and achievable; functional-focused goal statements and a mixture of short- (3–6 month) and long-term (9–18 month) goals are recommended. These can be grouped into symptomatic, passive function, active function, involuntary movement, and global mobility.

Clinical evaluation tools, goal setting, and outcome expectations for the multipattern treatment of shoulder spasticity with BoNT-A should be defined by the whole multidisciplinary team, ensuring patient and caregiver involvement.

**Discussion:**

These recommendations will be of benefit to clinicians who may not be experienced in evaluating and treating spastic shoulders.

## Introduction

Botulinum neurotoxin type A (BoNT-A) is an established first-line treatment in focal, multifocal, and segmental spasticity to provide pain relief, to reduce involuntary movements, and to help restore both passive and active functions (1–3). Most pivotal trials with BoNT-A in the upper limb (UL) spasticity did not include the injection of shoulder muscles (4–9), although a few recent trials reported the benefits of including shoulder muscles as possible injection targets (10, 11).

Injecting shoulder muscles in addition to the upper arm and other UL muscles represents a recent important change in the multidisciplinary management of UL spasticity with BoNT-A, and there have been some concerns over the difficulty of accurate injection, depending on the technique used. A group of experts convened to pool their knowledge of UL spasticity and BoNT-A injections to increase the awareness of why shoulder injection with BoNT-A should be considered by injectors, particularly those who may not be familiar with treating shoulder muscles, and to provide guidance on approaches to injection techniques and choice of muscle as well as goal setting. The output of their meetings is presented here and in an accompanying manuscript.

In the first manuscript, structural and functional anatomy, sensorimotor control, and control of spastic patterns, as well as synergies, were covered in the context of upper motor neuron syndrome and spasticity following traumatic brain injury, spinal cord injury, multiple sclerosis, or stroke. Recommendations on which shoulder muscles to inject were given. The importance of goal setting within a multidisciplinary team environment was explored, with recommendations that goal setting should be patient-centric and include both individualized short- and long-term goals. In this accompanying manuscript, we address BoNT-A injection technique and choice of outcome measurement scales, as well as provide case studies, to demonstrate how this guidance can be applied in practice.

## Methods

A two-part expert meeting was held online *via* video conference calls in October and November 2021 (3 h per video conference call). Five European experts who are members of university and teaching hospitals and national and international medical advisory boards in physical medicine and rehabilitation and neurological rehabilitation associations with a cumulative experience of more than 100 years in post-stroke spasticity gave focussed presentations on shoulder spasticity and treatment with BoNT-A injections followed by discussion.

Topics included structural and functional anatomy, synergies, goal setting, injection techniques, and case studies. To maximize the productivity of the meeting, pre-meeting surveys were conducted to capture information on each expert's preferred treatment practices. The surveys were drafted on behalf of the sponsor and included open-ended questions to drive the discussion sections of the meeting. The expert panel was asked to identify which patterns of UL spasticity are most commonly encountered in their daily practice, which shoulder muscles they treat for each pattern, and which activities are impaired by the most common and rare shoulder spasticity patterns following stroke. The panel were also asked for their top short- and long-term goals for the treatment of UL spasticity with shoulder involvement and recommended clinical evaluation scales.

The discussion was conducted in a way to identify consensus between treatment practices and to provide treatment recommendations based on this consensus. The online meetings were held with the intention of producing two linked manuscripts to present the findings as a practical guide for treating shoulder spasticity for the international neurorehabilitation community. In this second manuscript, case studies from the authors are provided to demonstrate the real-world application of this two-part practical guide.

## Results

### Outcome assessment, injection techniques, and adjuvant treatments

#### Outcome measurement scales

##### Clinical evaluation

Spasticity is complex, comprising more than a velocity-dependant increase in muscle tone (12); therefore, many factors need to be considered when clinically assessing UL spasticity. The expert panel recommendations on clinical assessment scales for UL spasticity included a combination of impairment and functional measures covering passive and active function of the UL, which can be used for setting and assessing goals. In this way, changes following intervention and the extent of those changes can be measured to allow for adjustments and optimization of both interventions and outcomes.

Range of motion (passive and active) should be assessed before treatment and quantified every time a patient exhibits a decrease in motion in the joint(s) of interest, as identified in a patient-centered goal (1).

The original Ashworth Scale (AS) was developed to assess the velocity-dependent increase in muscle tone in patients with multiple sclerosis by measuring resistance to passive stretching on a five-point (0–4) scale (13, 14). The AS was adapted to the six-point modified AS (MAS) in 1987 (15), and this scale is widely used to measure muscle tone in spasticity resulting from acquired brain damage, stroke, or spinal cord injury. The increased muscle tone that limits movement and imposes bad posturing in spasticity can be monitored over time using the MAS to measure the clinical impact of therapeutic interventions such as BoNT-A injections, occupational therapy, and other treatment modalities (e.g., kinesiotherapy and physical agents) (16, 17).

Inter-rater reliability of the MAS has been reported to be higher in UL than in lower limb assessment (17). In addition, the MAS demonstrated adequate test–retest reliability for the shoulder extensor and internal rotator muscles (18). The MAS has been shown to be responsive for detecting changes in muscle tone in patients with stroke; minimal clinically important differences of 0.48 and 0.76 in the UL muscles have been reported for effect sizes of 0.5 and 0.8 standard deviations, respectively (19).

More recently, the AS has also been adapted into a multi-dimensional score for the shoulder, as the AS shoulder sumscore (AS-SSS). The AS-SSS is the sum of AS scores for shoulder adduction, extension, and internal rotation (11, 20) and follows the principles of the summary rating scale for Resistance to Passive Movement (REPAS) (21). REPAS covers spasticity distribution over the body (focal, multifocal, segmental, multisegmental, and generalized spasticity) by providing regional body sub-scores and a total body score and was developed to be more reliable than the MAS by providing detailed guidelines on how to conduct the test (21). Due to the increased reliability over MAS, coupled with the shoulder-specific nature of the scale, AS-SSS is recommended for the assessment of spasticity in patients whose treatment goals are best quantified by overall tonus change in the shoulder.

For the assessment of spasticity-associated pain in the UL, the panel recommended the widely used numerical pain rating scale (22) or visual analog scale for pain (23), as well as the UL-specific Spasticity-Associated Arm Pain Scale (SAAPS). The SAAPS is an important way of communicating how proximal treatment improves spasticity-associated pain of the shoulder/arm/hand/fingers and function to the multidisciplinary team and the patient. The scale is used to collect data on the verbal/physiological pain response to a passive range of motion in five-arm segments, the first two of which include the shoulder. The validity and reliability of the SAAPS have been confirmed for the assessment of pain reduction in post-stroke UL spasticity following treatment with BoNT-A (24).

Functional outcomes for the UL (both passive and active function) can be measured with the Arm Activity Measure (ArmA). The ArmA has separate subscales for passive and active function and was developed to better measure arm function in a way relevant to real-life situations. The scale has been shown to be feasible for use in clinical practice and has a low burden on patients, carers, and clinicians (25).

In clinical practice, functional goals do not necessarily have to be measured by published scales. Discussions with the patient can include some specific goal(s) relevant to his/her life, which can be measured with a Likert-like scale or similar. For example, if the goal is to make it “easier” or “much easier” to wash the axilla with the unaffected upper limb, a four-point Likert-like scale can be used, such as 1 = more difficult than before; 2 = the same as before; 3 = easier than before; and 4 = much easier than before.

##### Goal attainment

Monitoring attainment of individualized Specific, Measurable, Achievable, Realistic, Time bound (SMART) goals can be managed by using the GAS or GAS-eous (Goal Attainment Scaling Evaluation of Outcome for Upper-limb Spasticity) tool (26). Goal setting is essential when treating patients with spasticity, and all goals should be patient-centric, with involvement from the patient or their family/caregiver in decisions regarding what is to be achieved and in what timeframe (1). Goal setting is discussed in more detail in the first manuscript of this series.

The Upper Limb Spasticity Index (ULSI) provides recommendations for relevant assessments and their incorporation into the characterization of the patient, goal setting, and treatment strategies, and ultimately how they reflect the patient's quality of life as related to UL spasticity (27). The ULSI includes three overall components: (i) severity and confounders to recovery (history and examination to characterize the patient and severity of impairment, including MAS); (ii) goals for treatment (utilizing individualized goal attainment scaling; GAS and GAS-eous); and (iii) standardized measures to assess different aspects of spasticity (including ArmA) (28). The ULSI was developed to provide a “brief battery” of assessment methods to cover diversity in the range of UL spasticity patterns, goals, and benefits of treatment (27).

Goals must be patient-centric and may include both short- and long-term aspirations to be achievable, demonstrate improvement, and allow time for the multimodal therapeutic approach to have an effect.

In a recent pooled analysis, repeated incobotulinumtoxinA injections were demonstrated to be effective in reducing UL pain associated with spasticity regardless of baseline pain severity and showed a cumulative effect over time with a greater effect on pain observed after multiple injection cycles (2). When BoNT-A is introduced early after the onset of spasticity, pain reduction is rapid, being observed after a single BoNT-A injection (29). However, maximal efficacy in passive and active function in most patients comes later, after three to four injections (30).

There may be a lag in efficacy for active versus passive function after BoNT-A injections in some cases due to the need for the body to adapt to disrupted synergies in motor control following a stroke or other cause of spasticity, and patients need to learn and practice their new abilities, which become possible after the effect on muscle tone is obtained. Neural pathways must be re-established; then, the patient needs to “relearn” how to move the UL. Injections with BoNT-A may free a trapped arm, restoring passive movement, but restoration of active movement requires active training as an adjuvant treatment. Such adjuvant treatments include task-oriented motor training, or self-guided rehabilitation, as well as additional functional electrical stimulation (FES) to relearn active movements. This also highlights the pertinence of including short- and long-term goals.

#### Injection techniques

##### Patient positioning

To aid the injection of BoNT-A, patient positioning is important. Although unassisted injection of superficial shoulder muscles can be done in practice using anatomical landmarks, guided techniques are particularly recommended for this muscle group due to the difficulty of accurate injection, particularly in deeper muscles (31) and in trunk muscles for risk of penetration into deeper structures like the pleura.

When injecting the ventral shoulder and upper arm muscles (e.g., the pectoralis major, the biceps brachii, and the anterior deltoid muscles), the patient should be placed in front of the injector in a stable upright position in a chair or in a supine position with neutral rotation on a stretcher. An anterior approach should be taken, from lateral to medial, in all cases of ventral shoulder and upper arm muscle injection (32).

For all other shoulder muscles (e.g., the posterior deltoid, the teres major, the latissimus dorsi, and the triceps brachii muscles), a dorsolateral approach in a sitting position or a lateral decubitus position on a stretcher should be used with the shoulder flexed. A dorsolateral approach should be taken, from lateral to medial.

For a subscapularis anterior approach (lateral to medial), a supine position should be used, with the shoulder in abduction (as much as possible, but usually 60–90 degrees). This is a more painful position for spastic patients. For a subscapularis posterior approach, a lateral decubitus position should be used with the shoulder flexed (32, 33).

##### Injection guidance and location

**Figure 1:**
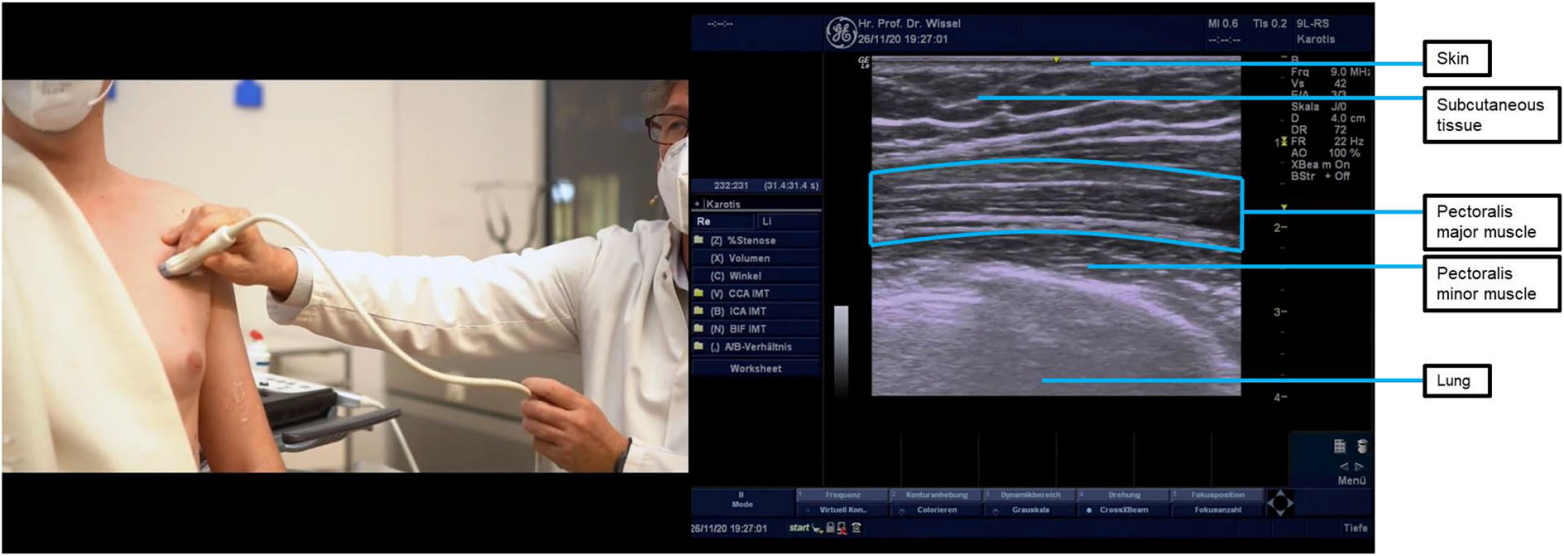
Ultrasound probe placement and image of the pectoralis major muscle. The pectoralis major muscle is shown at the top of the ultrasound image separated by white “rips” (this fibrillar pattern indicates that the probe and muscle fibers are parallel to each other), and the pleura is shown at the bottom. Asking your patient to breathe in and out helps to identify the pleura which must be avoided when injecting. Turning the ultrasound probe through 90 degrees reveals the different fiber orientation of the pectoralis major and pectoralis minor muscles (pectoralis minor shown in the middle of the ultrasound image, which has small white dots—a starry sky appearance—indicating that the probe is somewhat perpendicular to the direction of muscle fibers.

**Figure 2:**
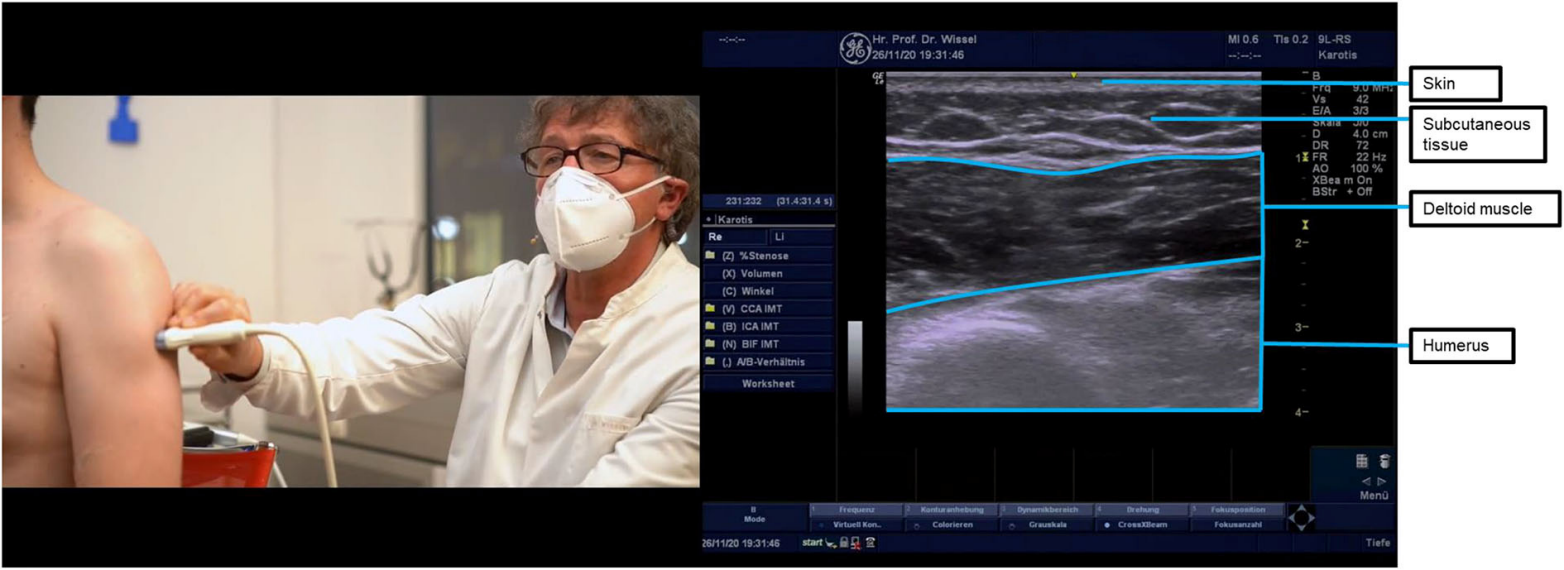
Ultrasound probe placement and image of the intermediate deltoid (pars acromialis) muscle. The deltoid has a monomorphic appearance with stripes, when the ultrasound probe is placed longitudinally, and dots with facias/tendon-like structures, when the probe is transversally orientated. The injection target to treat retroversion and extension is the posterior part of the deltoid muscle. In the figure, the probe is compressing the arm to show the deltoid muscle at the top and humerus muscle at the bottom of the ultrasound image. To visualize and inject the anterior deltoid, the probe should be slid anteriorly from the position shown; to visualize and inject the posterior deltoid, the probe should be slid posteriorly.

**Figure 3:**
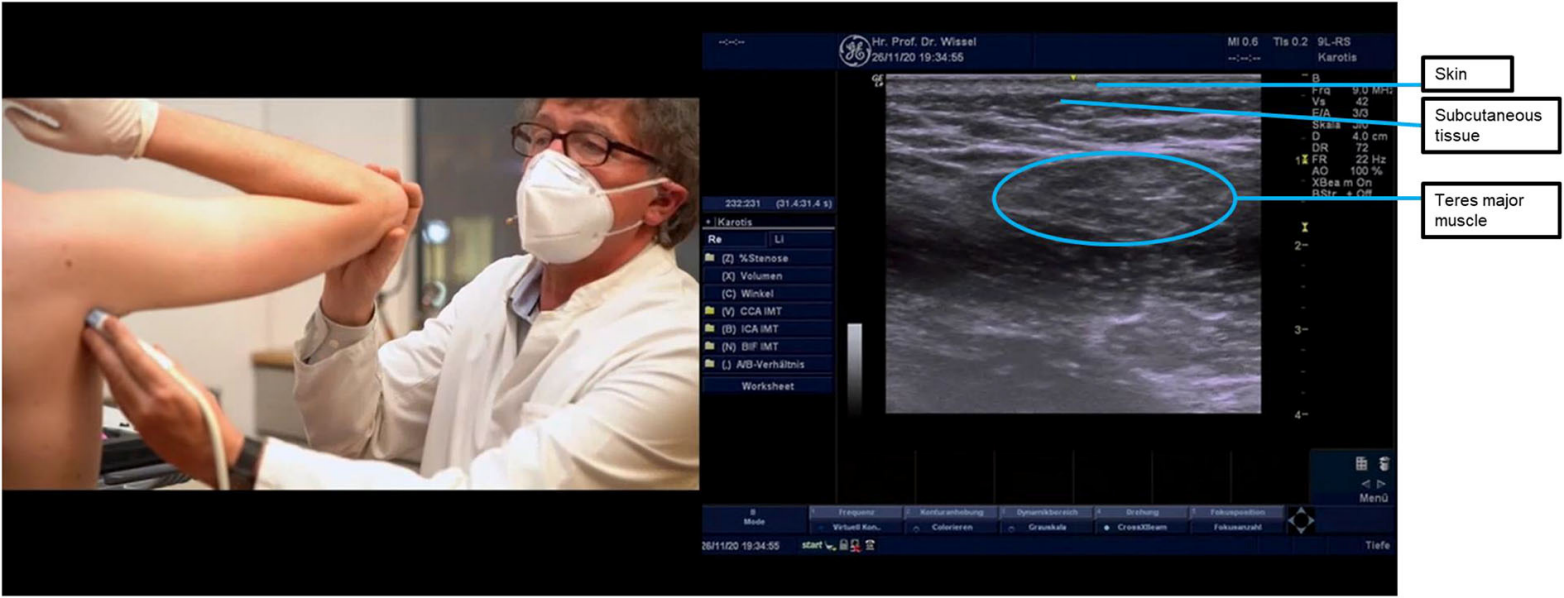
Ultrasound probe placement and image of the teres major muscle. The round muscle in the middle of the ultrasound image is the teres major muscle. The ultrasound probe can be turned through 90 degrees to help identification, with the teres major muscle having a striped appearance when the ultrasound probe is placed longitudinally.

**Figure 4:**
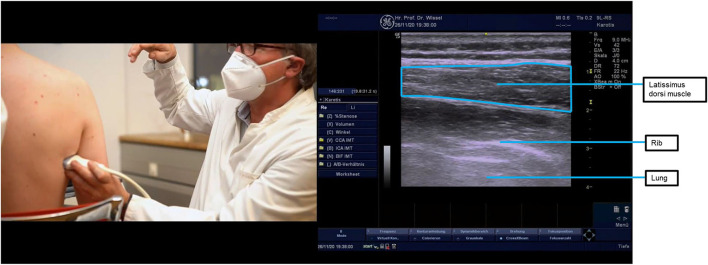
Ultrasound probe placement and image of the latissimus dorsi muscle. The latissimus dorsi muscle can be located by beginning at the posterior axillary fold and then moving the ultrasound probe down the thorax, about halfway toward the lower thoracic vertebrae. To better distinguish the muscle from the subcutaneous tissue, the probe should be parallel to muscle fiber orientation, thus making its fibrillar pattern apparent. In this anatomical location, the latissimus dorsi will be the most superficial muscle, as shown in the image. Underneath it lies the serratus anterior muscle, also shown in the ultrasound image. Just under the serratus anterior muscle, it is also possible to see one rounder hyperechoic line on the bottom right, which corresponds to a rib, and a more linear hyperechoic line on the bottom left, which corresponds to the pleura. The injection can be performed with the probe either in the longitudinal or transversal orientations, but longitudinal is preferred due to the proximity of the pleura. Multiple injections (two or three) are recommended for the latissimus dorsi due to the size and strength of the muscle.

**Figure 5:**
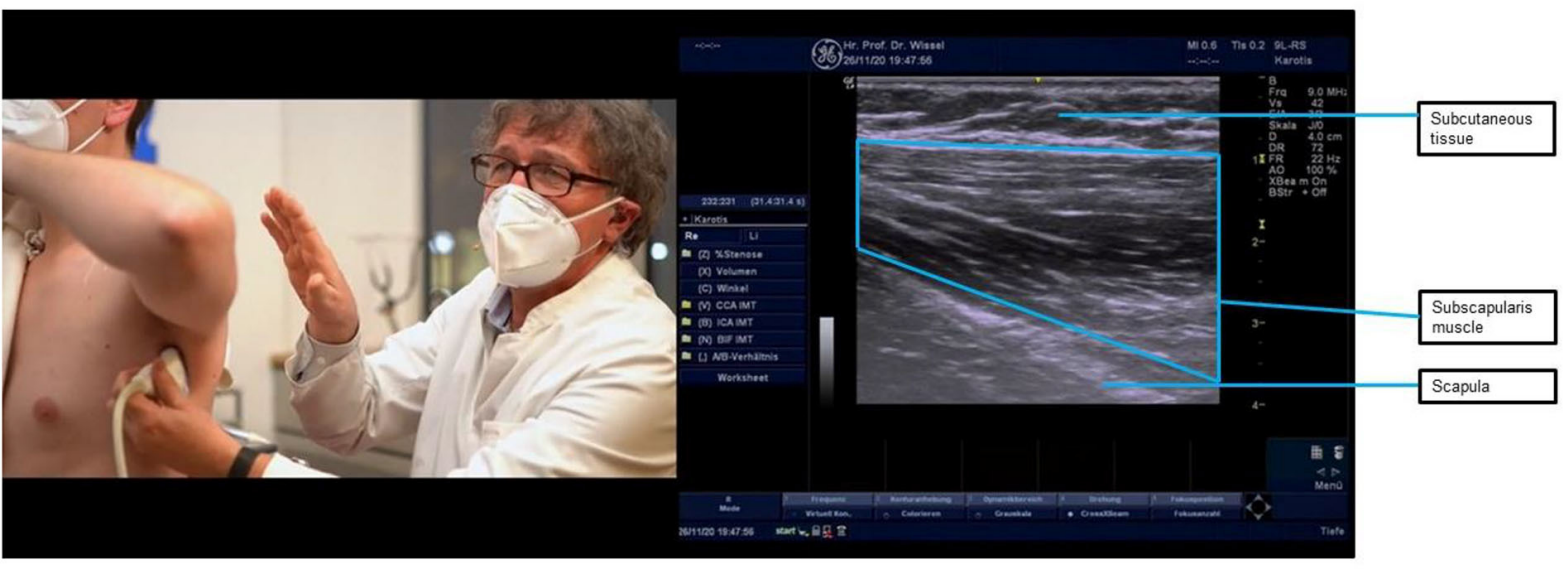
Ultrasound probe placement and image of the subscapularis muscle. The subscapularis is the strongest inner rotator of the shoulder muscles. As the subscapularis muscle is located under the shoulder blade, ultrasound location requires elevation of the arm with probe placement in the axilla pointing toward the shoulder blade. The subscapularis muscle is visible as the striped structure in the ultrasound image, below the subcutaneous tissue at the top of the image. Injection of the subscapularis muscle requires two people—one to hold the arm while the patient is lying supine and one to hold the ultrasound probe and inject. Injecting behind the probe location shown above, aiming away from the thorax and toward the scapula, is the only safe approach as the ultrasound probe protects the thorax, and on the other side of the subscapularis lies the scapula, which cannot be harmed when injecting.

An injection guidance technique is always preferred to an injection based only on anatomical landmarks. The guidance method most recommended by the expert panel for injecting the shoulder was ultrasound imagery (Figures 1–5). Electromyography (EMG) and electrical stimulation are also possible methods for identifying the muscles to inject but are not as accurate as ultrasound in our experience and that of others (34–37). In the case of electrical stimulation, this can be more painful for the patient due to a larger needle size and the need for electrical impulses. Also, frequently more than one skin puncture is needed per injection site to find the best location using electrical stimulation; with ultrasound guidance, it is often possible to inject more than one muscle with only one skin puncture.

Injection guidance, in particular with ultrasound, increases precision in muscle targeting, which may lead to improved effectiveness and reduced costs (better outcome from the same dose), and reduced risk of adverse events that may result from the inadvertent injection of neighboring muscles or the injuring of nerves or vascular structures in the vicinity of the target muscles (33).

Ultrasound was recommended by the expert panel as the optimal method for guiding BoNT-A injection around the shoulder. It allows clear visualization of the different structures around the target muscle, as well as the target muscle itself. Visualization occurs in real time, meaning that altered anatomy can be visualized, which may not be the case when only anatomical landmarks, EMG, or electrical stimulation are used without other guidance. Overlaying muscles can be visualized, which is of particular importance in the shoulder due to the way they are organized. Ultrasound can increase the speed of injection as multiple target muscles can be quickly identified and injected during the same needle insertion (38).

#### Adjuvant treatments

Adjuvant treatments after BoNT-A injection include a variety of measures applied after injection in a multidisciplinary team setting (e.g., physical and/or occupational therapy, splinting, orthotics, and all forms of exercises). They may also involve serial adhesive taping and casting, extracorporeal shockwave therapy, electrical stimulation, and whole-body vibration therapy (39). Data from a recent meta-analysis—as well as clinical guidelines—recommend adjuvant treatments after BoNT-A injection, mainly using joint posture procedures (1, 40). There are currently no published papers on such treatments in patients with shoulder muscle spasticity, and some treatments, such as taping and casting, are not feasible in this anatomical region. The only treatments that have been described (in publications) after BoNT-A injection in shoulder muscles are transcutaneous electrical nerve stimulation (41) and constraint-induced movement therapy (42). However, the addition of passive mobilization, shoulder posturing, and, when possible, task-oriented active physiotherapy and occupational therapy were recommended by the expert team.

### Patient cases—shoulder treatment

#### Case 1

A 52-year-old female patient with a prior history of arterial hypertension experienced an ischemic stroke of the left cerebral hemisphere in 2011. In 2016, a physical examination revealed right hemiplegia, with no active UL muscle contraction, right hemibody spasticity-related pain—especially upon mobilization of the shoulder, elbow, and fingers—and right hemibody mechanical allodynia, especially when rubbing garments like a bath towel. Muscle hypertonia, pain upon mobilization, and mechanical allodynia are graded in Table 1. The shoulder presented a spastic pattern in adduction and internal rotation. Treatment with incobotulinumtoxinA was suggested. The case is summarized in Table 2.

**Table 1:** Case reports: Baseline measurements.

**Case 1**	
MAS score	
Shoulder adductors	3
Shoulder internal rotators	3
Shoulder extensors	3
Elbow flexors	3
Pain (NRS)	
Shoulder	8/10
Elbow	7/10
Hand	8/10
Mechanical allodynia on shoulder	8/10
**Case 2**	
MAS scor	
Shoulder abduction	3
Shoulder flexion	3
Shoulder external rotation	3
Elbow extension	2
Pain (VAS)	
Shoulder abduction	8
Shoulder flexion	8
Shoulder external rotation	8
Elbow extension	4
Wrist extension	2
Finger flexion	10
Thumb terminal phalanx flexion	8
**Case 3**	
MAS score	
Shoulder flexion	3
Shoulder external rotation	3

MAS, Modified Ashworth Scale; NRS, numerical rating scale; VAS, visual analog scale.

**Table 2:** Case 1: A 52-year-old female patient with prior history of arterial hypertension who experienced an ischemic stroke of the left cerebral hemisphere that caused a right spastic hemiplegia and was a candidate for botulinum toxin injections.

**Assessment**	**Result**
Physical exam	Right hemiplegia, with no active muscle contraction in the UL, Spastic hypertonia of several shoulder, arm, forearm, and hand muscles. The shoulder presented a spastic pattern in adduction and internal rotation
Pain	Right hemibody spasticity-related pain in the shoulder, elbow, and hand that was worse upon mobilization. Right hemibody mechanical allodynia, especially when rubbing garments such as a bath towel
Goal setting (using GAS)	Short-term goals: To improve pain upon mobilization of the shoulder from 8/10 to 0/10 at the week 4 assessment post-each injection cycle.^a^ To improve mechanical allodynia in the right upper limb when rubbing a bath towel from 8/10 to 1/10 at the week 4 assessment post-each injection cycle^a^
Treatment	IncobotulinumtoxinA injections were administered as shown in Table 3. Treatment was selected for the following reasons: the patient presented with a spastic pattern in adduction and internal rotation. The pectoralis major is the main shoulder adductor, and hence it was chosen. The latissimus dorsi is also an important adductor and internal rotator, and was chosen because the patient also presented with hypertonia in the extensor group. The subscapularis was included because the patient continued presenting resistance to external rotation even with the shoulder in a passively adducted and extended position (suggesting that the subscapularis was also responsible for the internal rotation component since, when in that position, the latissimus dorsi and the teres major are in a shortened position, theoretically lessening resistance to passive movement)
Reassessment (using GAS)	At 4 weeks, the patient had achieved her short-term (recurrent) goals: To improve pain upon mobilization of the shoulder from 8/10 to 0/10 at the week 4 assessment post-each injection cycle: GAS score = 0 (pain graded as 0/10, no pain). To improve mechanical allodynia in the right upper limb when rubbing a bath towel from 8/10 to 1/10 at the week 4 assessment post-each injection cycle: GAS score = 0 (pain graded as 1/10). As expected, the patient has been attaining these ongoing goals since starting injections with incobotulinumtoxinA in 2016; she received regular injections every 3–4 months, and has completed 17 injection cycles to date
Adjuvant therapy	Recommended after each injection; however, not actioned by the patient

a The patient's initial goal was to reduce pain from 8/10 to 4/10; however, she experienced such dramatic improvement (GAS +2) that the treatment goals for each injection cycle were reset as indicated here.

GAS, goal attainment scaling; UL, upper limb.

Short-term treatment goals were set to be measured after the first injection cycle of botulinum toxin; adjustment of the pre-treatment goals was necessary because of initial rapid and dramatic improvement after the first injection cycle. The primary goals were to improve pain upon mobilization of the shoulder and to improve mechanical allodynia in the right upper limb when rubbing a bath towel.

Treatment comprised of injections of incobotulinumtoxinA into multiple muscle patterns in the shoulder, the arm, the forearm, and the hand under ultrasound guidance (Table 3 shows the doses injected into each muscle). These muscles were selected because the patient presented with a spastic pattern in adduction and internal rotation. The pectoralis major is the main shoulder adductor—hence, it was chosen. The latissimus dorsi is also an important adductor and internal rotator and was chosen because the patient also presented with hypertonia in the extensor group. The subscapularis was included because the patient presented continued resistance to external rotation even with the shoulder in a passively adducted and extended position (suggesting that the subscapularis was also responsible for the internal rotation component since, when in that position, the latissimus dorsi and the teres major are in a shortened position, theoretically lessening resistance to passive movement). Ultrasound probe placement for imaging these muscles is shown in Figures 1, 4, 5.

**Table 3:** IncobotulinumtoxinA injection schedule for each case report.

**Location**	**Muscle**	**Dose (Case 1)**	**Dose (Case 2)**	**Dose (Case 3)**
Shoulder	Subscapularis	50 U (2 injection points)	50 U (1 injection point)	–
	Latissimus dorsi	50 U (2 injection points)	70 U (2 injection points)	100 U (2 injection points)
	Pectoralis major	50 U (2 injection points)	50 U (2 injection points)	–
	Teres major	–	40 U (1 injection point)	75 U (2 injection points)
	Deltoideus posterior	–	–	50 U (1 injection point)
Arm	Biceps brachii	50 U (2 injection points)	–	–
	Brachialis	50 U (2 injection points)	–	–
	Brachioradialis	25 U (1 injection point)	40 U (1 injection point)	–
Forearm	Flexor digitorum superficialis	50 U (2 injection points)	75 U (2 injection points)	–
	Flexor digitorum profundus	25 U (1 injection point)	75 U (2 injection points)	100 U (2 injection points)
	Flexor pollicis longus	50 U (2 injection points)	40 U (1 injection point)	–
Hand	Flexor pollicis brevis	–	–	25 U
	Opponens pollicis	10 U (1 injection point)	–	30 U (1 injection point)
Total upper limb dose		410 U	440 U	380 U
Total shoulder dose		150 U	210 U	225 U

After 4 weeks, the patient was re-assessed and was found to have achieved her short-term goals. Specifically, the goal to improve pain upon mobilization of the shoulder at the week 4 assessment after each injection cycle was achieved. The goal to improve mechanical allodynia in the right upper limb when rubbing a bath towel was also achieved. As was expected, the patient has been attaining these ongoing goals since starting injections with incobotulinumtoxinA in 2016; she received regular injections every 3–4 months and has completed 17 injection cycles up to 2022. By maintaining the injection interval and doses, it was possible to avoid loss of treatment benefits, hence avoiding the rollercoaster experience. The patient has been advised to take part in adjuvant therapy after each injection visit but has been unable to do so for family reasons.

#### Case 2

A female patient in her 60s presented 12 years after traumatic brain injury. Acute brain imaging had shown bifrontal and left temporal haemorrhagic contusions and traumatic subarachnoid hemorrhage, as well as edema with midline shift. The patient was treated acutely with decompressive craniectomy and subsequent cranioplasty. Sub-acute brain imaging showed a signal change in the left cerebral peduncle, and a scan at 3 years post-injury showed atrophy, extensive left frontotemporal gliosis, and Wallerian degeneration of the left internal capsule and cerebral peduncle. After a decade of stability, with no significant tone issues and no regular anti-spasticity medication despite some right hemiplegia, the patient developed a rapidly evolving pattern of tone throughout the UL and the shoulder. This tone progressed unusually rapidly and was maximal proximally and distally.

Carers estimated that the onset to time to referral into the spasticity service was <3 months. The referral was expedited as urgent by the patient's primary care doctor as the reduction in passive range was making personal care and positioning painful and threatening axillary and palmar hygiene and skin integrity. Opioid analgesia had been prescribed. On physical examination, the shoulder was adducted, extended, and internally rotated, and passive movement was not possible. Attempts to move were anticipated by pain responses. There was increased tone throughout elbow movement, but passive movement was possible to −20 degrees with a catch at two-thirds range sustained after. The hand was in tight mass flexion with a flexed terminal phalanx of the thumb that had resulted in skin breakdown because the thumbnail was rubbing against the first phalanx of the index finger. The use of an inflatable “carrot” splint was not successful, and when the patient was last able to obtain some passive finger movement, carers observed palmar skin breakdown. This breakdown of the skin was expected to require surgical intervention. Baseline measurements are shown in Table 1, and the case is summarized in Table 4.

**Table 4:** Case 2: A female patient in her 60s who presented 12 years after traumatic brain injury with a rapidly evolving pattern of tone throughout the UL and shoulder and was a candidate for botulinum toxin injections.

**Assessment**	**Result**
Physical exam	Shoulder was adducted, extended and internally rotated, and passive movement was not possible. Increased tone throughout elbow movement, but passive movement was possible to −20 degrees with catch at 2/3 range sustained after. The hand was in tight mass flexion with flexed terminal phalanx of thumb that had resulted in skin breakdown because the thumb nail was rubbing against the first phalanx of the index finger
Pain	Attempts to move were anticipated by pain responses
Goal setting (using GAS)	Short-term goals: To reduce pain in washing, dressing, and positioning an inflatable “carrot” splint. To allow cleaning of the palm and axilla. To improve ease for carers washing and dressing the patient. Long-term goal: To avoid the need for more invasive procedures
Treatment	IncobotulinumtoxinA injections were administered as shown in Table 3. All muscles producing shoulder adduction and internal rotation were targeted, with the aim of maximizing the passive range. Reducing proximal tone was hoped to reduce drive to proximal and distal pain responses
Reassessment	At 3 weeks, the patient had achieved her short-term (recurrent) goals: Washing and dressing without the requirement for additional analgesia possible. Dramatically improved ease of personal care for carers. Sufficient distal passive range achieved to make splint use consistent and protect the palmar skin. The need for surgical intervention was avoided, thereby the long-term goal was achieved
Adjuvant therapy	Inflatable “carrot” splint to unclench hand

GAS, goal attainment scaling; UL, upper limb.

The goals for this patient were set and included the short-term goals to reduce pain, improve hygiene, and improve ease for carers; and the long-term goal was to avoid the need for more invasive procedures.

Under ultrasound guidance (Figures 1, 3–5), injections of BoNT-A (posterior approach to subscapularis) were made into the shoulder, arm, and forearm muscles at the locations and dosages indicated in Table 3. Targeting all muscles producing shoulder adduction and internal rotation aimed to maximize the passive range, while it was hoped that reducing proximal tone would reduce the drive to proximal and distal pain responses.

Upon reassessment, greater than expected significant benefit with respect to the pain-free range of proximal movement was observed 3 weeks post-injection. This allowed washing and dressing without the requirement for additional analgesia and dramatically improved the ease of personal care for carers. The sufficient distal passive range was achieved to make splint use consistent and protect the palmar skin, thereby avoiding the need for surgical intervention. The proximal effect and reduced pain were likely synergistic in reducing distal motor drive.

#### Case 3

A 42-year-old female patient with marantic endocarditis in anti-phospholipid syndrome and labile international normalized ratio (INR) under oral anticoagulation with acenocoumarol experienced a right-sided cerebellar and thalamic stroke in February 2007, resulting in slight left-hand hemiparesis. This was followed by a right-hand pontine stroke in July 2007 that resulted in further left-hand hemiparesis. In July 2014, she experienced multiple strokes with cortical atrophy (memory, attentional, and executive deficit) and secondary seizures. The patient then presented in 2019, 12 years after the first stroke and 5 years after the most recent stroke, with the appearance of left thumb-in-palm deformity and adducted shoulder. She was injected with incobotulinumtoxinA to the left arm in May 2019 but was then lost to follow-up. The patient re-presented in September 2020, showing a recurrence of thumb-in-palm deformity and clenched fist, with a painful, adducted shoulder. She had a deficit in shoulder flexion and abduction.

On physical examination, the left shoulder had a maximal active elevation of 65 degrees and a maximal active abduction of 36 degrees. The patient had difficulties with hygiene, with pain on passive mobilization, and thumb in palm with severe pain during any attempt of mobilization. Probable contracture of the flexor pollicis brevis was noted.

Goal setting resulted in a primary goal of shoulder hygiene. The secondary goal was pain reduction at the shoulder and hand. Treatment was initiated using multiple injections of incobotulinumtoxinA into the shoulder, forearm, and hand patterns (Table 3; the case is summarized in Table 5). Ultrasound probe placement for the target muscles is described in Figures 2–4.

**Table 5:** Case 3: A 42-year-old female patient who, after a series of strokes between 5 and 12 years prior, initially presented with the appearance of left thumb-in-palm deformity and adducted shoulder, was treated with incobotulinumtoxinA and then lost to follow-up.

**Assessment**	**Result**
Physical exam	Left shoulder maximal active elevation was 65 degrees and maximal active abduction 36 degrees. Probable contracture of the flexor pollicis brevis was noted
Pain	Patient had difficulties with hygiene, with pain on passive mobilization. Thumb-in-palm deformity with severe pain during attempt of mobilization
Goal setting (using GAS)	Primary goal: Shoulder hygiene, to be able to wash her own armpit. Secondary goal: Pain reduction at the shoulder and hand
Treatment	Treatment was initiated using multiple injections of incobotulinumtoxinA into shoulder, forearm and hand patterns, as shown in Table 3
Reassessment	At 5 weeks, the patient had achieved her primary and secondary goals: Shoulder had active elevation of 115 degrees, passive elevation of 140 degrees, and painless external rotation of 40 degrees. Examination of the hand showed that active opening was possible, the thumb had −40 degrees passive extension (I metacarpophalangeal joint) and −70 degrees active extension. The patient was able to wash her armpit alone, without pain, and could actively open her hand without pain and clean her hand
Adjuvant therapy	Physiotherapy (passive mobilization of the shoulder and the hand), twice a week

The patient re-presented, over a year after initial presentation, with a recurrence of thumb-in-palm deformity with a clenched fist and painful, adducted shoulder.

GAS, goal attainment scaling.

Reassessment 5 weeks after treatment revealed that the shoulder had an active elevation of 115 degrees, passive elevation of 140 degrees, and painless external rotation of 40 degrees. Examination of the hand showed that active opening was possible; the thumb had −40 degrees passive extension (I metacarpophalangeal joint) and −70 degrees active extension. The patient was able to wash her armpit alone, without pain, as expected. She could also actively open her hand without pain and clean her hand, better than expected.

## Discussion

In part 2 of this practical guide, we have provided recommendations on clinical evaluation tools, described guidance techniques for injection of BoNT-A in the shoulder muscles, and presented case studies to demonstrate how indications from both parts of the guide can be put into practice to improve patient outcomes. This guide is intended to highlight the importance and benefits of treating the shoulder muscles in UL spasticity and to help practitioners who need to treat these patients.

## Data availability statement

The datasets presented in this article are not readily available because of ethical and privacy restrictions. Requests to access the datasets should be directed to: JW, joerg@schwarz-wissel.de.

## Ethics statement

Ethical review and approval was not required for the study on human participants in accordance with the local legislation and institutional requirements. The patients/participants provided their written informed consent to participate in this study. Written informed consent was obtained from the individual(s) for the publication of any potentially identifiable images or data included in this article.

## Author contributions

All authors were involved in the conception and choice of approach for the expert consensus, contributed findings from their knowledge and experience to the expert consensus, drafted the manuscript and critically revised it for important intellectual content, and approved the final version of the manuscript.
